# Evaluation of the implementation of multidisciplinary fast-track program for acute geriatric hip fractures at a University Hospital in resource-limited settings

**DOI:** 10.1186/s12877-021-02509-y

**Published:** 2021-10-12

**Authors:** Unchana Sura-amonrattana, Theerawoot Tharmviboonsri, Aasis Unnanuntana, Direk Tantigate, Varalak Srinonprasert

**Affiliations:** 1 Department of Medicine, Division of Geriatric Medicine, Faculty of Medicine Siriraj Hospital, Mahidol University, Bangkok, Thailand; 2 Department of Orthopedic Surgery, Faculty of Medicine Siriraj Hospital, Mahidol University, Bangkok, Thailand

**Keywords:** Hip fracture, Orthogeriatric, Elderly, Dementia, Delirium, Complications, Length of stay, Mortality

## Abstract

**Background:**

Hip fractures are common among frail, older people and associated with multiple adverse outcomes, including death. Timely and appropriate care by a multidisciplinary team may improve outcomes. Implementing a team to jointly deliver the service in resource-limited settings is challenging, particularly on the effectiveness of patient outcomes.

**Methods:**

A retrospective cohort study to compare outcomes of hip fracture patients aged 65 or older admitted at Siriraj hospital before and after implementation of the Fast-track program for Acute Geriatric Hip Fractures. The primary outcome was the incidence of medical complications. The secondary outcomes were time to surgery, factors related to the occurrence of various complications, in-hospital mortality, and mortality at month 3, month 6 and month 12 after the operation.

**Results:**

Three hundred two patients were enrolled from the Siriraj hospital’s database from October 2016 to October 2018; 151 patients in each group with a mean age of 80 years were analyzed. Clinical parameters were similar between groups except the Fast-track group comprising more patients with dementia (37.1% VS 23.8%, *p* < 0.012). In the Fast-track group, there was a significantly higher proportion of patients underwent surgery within 72-h (80.3% VS 44.7%, *p* < 0.001) and the length of stay was significantly shorter (11 days (8–17) VS 13 days (9–18), *p* = 0.017). There was no significant difference in medical complications. Stratified analysis by dementia status showed a trend in delirium reduction in both patients with dementia and without dementia groups, and a pressure injury reduction among patients with dementia after the program was implemented but without statistical significance. There was no significant difference in mortality.

**Conclusions:**

The implementation of a multidisciplinary team for hip fracture patients is feasible in resource-limited setting. In the Fast-track program, time to surgery was reduced and the length of stay was shortened. Other outcome benefits were not shown, which may be due to incomplete uptake of all involved disciplines.

## Background

Hip fractures are common injuries that result in loss of function, reduction in quality of life, an increase in morbidity and mortality in older people [1, 2]. Globally, more than 4.5 million patients per year suffer from medical complications due to improper management of hip fractures. As society ages, post-hip fracture morbidity may affect up to 21 million persons in 2050 [3–6]. Male gender, older age, and multiple comorbidities are associated with an increased risk of death within the first year after a hip fracture [7]. In Thailand, there is an increase in the incidence of hip fractures in people aged 65 years old and older, which substantially increased short term and long term mortality.

Most hip fractures are more likely to occur in frail older people with several geriatric syndromes such as functional impairment, malnutrition and dementia [8, 9]. Consequently, those patients with complex medical comorbidities require more attention prior to undergoing operation. Moreover, they are more likely to be at risk of postoperative complications and prolonged hospital stays with their pre-morbid complexity. Multiple studies [9–26] have reported that factors such as proper care at presentation, optimal pain control, an orthogeriatric model of care, comprehensive geriatric assessment (CGA), expedited time to surgery and early rehabilitation are associated with a lower risk of complications and decreased mortality in older patients with hip fractures.

Thailand is a middle-income country where proportion of older people has risen rapidly, reaching 18% of the population in 2021 [27]. However, the number of health care personnel specialized for taking care of older people is limited [28–30]. Allocation of human resources and operating rooms for the multidisciplinary care team for non-emergency condition such as hip fracture is challenging in resource-limited settings. After the existing literature had been reviewed [31–34] showing sparse evidence from resource limited settings, a multidisciplinary program titled “Acute Geriatric Hip Fracture: Fast Track in Siriraj Hospital” was initiated for older patients with hip fractures in September 2017. Initially, the team composed of orthopedic trauma team, geriatricians, anesthesiologists, physical therapists and nurse coordinators attempted to contemplate the flow for patient care according to international practice guidelines. The structure, process and outcome quality indicators in Table 1 have been set and continuously monitored through quarterly meetings. The primary target of the service team was to reduce the time to surgery, while the ultimate goal was to improve patients’ outcomes. The purpose of this study was to evaluate patient outcomes before and after implementation of the Fast-track program for Acute Geriatric Hip Fractures. The primary outcome was the incidence of medical complications. The secondary outcomes were time to surgery, causes of delayed surgery, short term and long term morbidity and short term and long term mortality.

**Table 1:** The structure, process and outcome quality indicators for the implementation of the Fast-track program for Acute Geriatric Hip Fractures

**Structure quality indicators**	Orthogeriatric management during admission
	Using an agreed multidisciplinary protocol
	Hip fracture surgery planned on an operation list
**Process quality indicators**	Assessed by a geriatrician within 24 h
	Assessed by the Acute Pain Service (APS) within 24 h
	Immediate analgesia on presentation and in case of pain
	Time to surgery within 72 h
	Early ambulation after surgery
**Outcome quality indicators**	Intraoperative adverse events
	Postoperative major medical complications
	Re-operation rate
	Length of hospital stay
	In-hospital mortality, 3-month mortality, 6-month mortality, 1-year mortality
	Discharge destination

## Methods

### Study design and data collection

All patients with hip fractures admitted to the Department of Orthopedics, Siriraj Hospital, Thailand, from October 2016 to October 2018 were identified retrospectively from the Siriraj hospital’s database. The database comprised medical records from inpatient and outpatient services. For inpatient documents, all progress notes documented by the multidisciplinary team were collected. The database also included electronic laboratory data and image data. The admitted hip fracture patients aged 65 years or older with complete medical records were selected to be the subjects for the study. Patients who underwent elective surgery were excluded, and then the included population were divided into the PRE-fast track group and the Fast-track one according to the time of the program implementation.

**Fig. 1:**
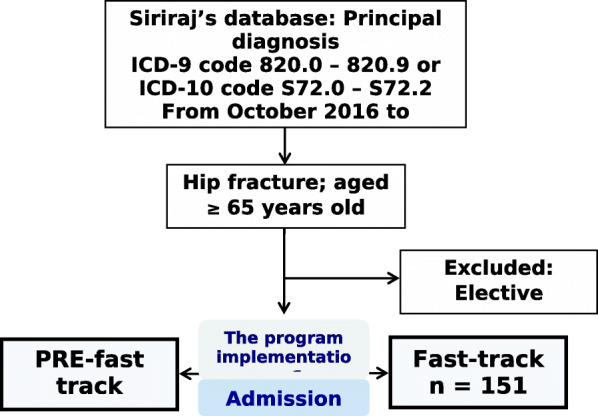
Subject selection flow chart

Medical records were retrieved according to the ICD-9,10 (International Classification of Diseases 9-10th Revision) diagnostic codes for hip fracture (820.0–820.9 and S720-S722). Among 905 medical records identified, 803 patients aged ≥65 years meeting inclusion criteria were included (Fig. 1). After having initially reviewed, medical records were sorted, according to the admission number (AN) for both the PRE-fast track group and the Fast-track one. Then, we started to review forward according to the admission number in the Fast-track group and backward in the PRE-fast track group from the day of the starting program until the sample size target was met.

All relevant medical records were reviewed to identify patients’ medical comorbidities, premorbid functional status, interventions and complications occurring during hospitalization. With respect to delirium, the detection of delirium was performed by a clinical trainee in geriatric medicine. The patient was determined to have delirium if the medical record contained any document representing awareness of the syndrome, progress notes describing delirium or confusion, notes attempting to identify the causes of delirium, or notes describing any treatment to control delirium symptoms. Discharge summaries were also reviewed to identify any document of the signs and delirium symptoms.

Beginning in July 2016, all hip fracture patients admitted to orthopedic wards were recruited in the Siriraj Fracture Liaison Service (FLS). Patients in the FLS registry were followed from hospital admission until discharge and subsequent visits. Information regarding mortality and functional outcomes was obtained through electronic hospital records and data from the FLS registry.

### PRE- the fast-track program for acute geriatric hip fractures (PRE-fast track program)

The orthopedic trauma team was responsible for the standard hip fracture treatment including pain control, basic preoperative assessment and scheduled surgery time. Surgery would be performed according to the availability of operative rooms and surgeons’ regular work schedules. Consultation with the on-call medical teams and the geriatric team was available on request. Physical therapists assessed the patients when they were admitted to the orthopedic ward, but there was no standard protocol on mobilization or postoperative medical management (Table 2).

**Table 2:** Comparison of the PRE-fast track program and the Fast-track program for Acute Geriatric Hip Fractures (Fast-track program)

PRE-fast track program	Fast-track program
- Managed by the orthopedic trauma team - Admitted to the orthopedic wards - Consult on-call medical teams - Geriatric teams available on request - Physical therapists assessed the patients when they were admitted to the orthopedic ward, but no a standard protocol on mobilization	- Reviewed by the orthopedic, geriatric and Acute Pain Service (APS) teams within 24 h of admission - Admitted to the orthopedic wards - Geriatric team review to optimize medical condition preoperative and monitor during admission - APS operated by anesthesiologists aims to optimize pain control within the first 24 h by providing femoral nerve catheter blockade - Surgery within 72 h required after admission - Physical therapists assessed the patient conditions on admission to the orthopedic ward; aimed to achieve at least a sitting position on the first postoperative day

### The fast-track program for acute geriatric hip fractures (fast-track program)

The Fast-track model is a multidisciplinary team of medical specialists and allied health teams including orthopedists, geriatricians, anesthesiologists, physical therapists and nurse coordinators. The Fast-track care commences on admission to the hospital. The role of each discipline in the team and assessment timeframe were assigned in the protocol. Acute pain service (APS), operated by anesthesiologists, aims to optimize pain control within the first 24 h by providing femoral nerve catheter blockade, and then customizing pain medications. Applying the orthogeriatric model of care as a framework, the geriatric team manages the patients within the first 24 h of admission until the patients were discharged from the hospital. Surgery is scheduled as quickly as possible, and spinal anesthesia is the preferable method. The Fast-track program aims to have all patients in the surgical operating room within 72 h of admission. An operating room is also dedicated for the hip fracture patients, which allows the patients to undergo surgery as planned. The program also aims to prevent medical complications (delirium, urinary tract infection, pressure injury, stroke, pulmonary embolism/deep vein thrombosis, myocardial infarction), provide nutritional counseling and supplements, achieve at least a sitting position on the first postoperative day, and begin the discharge planning in the early period of care (Table 2).

### Outcomes

The primary outcome was the incidence of medical complications. The secondary outcomes were the proportion of achieving the 72-h time-to-surgery target, causes of delayed surgery, in-hospital mortality, mortality at month 3, month 6, month 12 after the surgery and function status (*Barthel index*) at post-operative day 4, month 3 and month 12.

### Statistical analysis

For the comparison between the patient outcomes of the PRE-fast track program and those of the Fast-track program, a sample size calculation was conducted by assuming the incidence of delirium in the conventional group of 53 and 37% in the intervention group [35]. With 80% power and a 5%, 2-sided level of significance, the estimated sample size was 151 subjects per group.

Baseline characteristics and related factors were analyzed by using descriptive statistics. Categorical variables were analyzed by using Chi-square test. Fisher’s exact test was used for categorical data with a count of less than 5. All continuous data were tested for normality. Independent sample t-test and Mann-Whitney U test were used to compare continuous variables, depending on the data distribution. The *p*-value < 0.05 was considered statistical significance. Statistical analysis was performed by using SPSS for Windows version 18 software.

## Results

302 patients were enrolled from the Siriraj hospital’s database from October 2016 to October 2018; 151 patients in each group. The mean age of the total population was 80 years, and 43 (28.5%) of subjects were ≥ 85 years of age. Gender distribution, comorbidities, and the Charlson Comorbidity Index (CCI) between both groups were not statistically different. There was a higher proportion of dementia in the Fast-track group (37.1%) compared to the PRE-fast track group (23.8%) (*p* = 0.012). The mean BMI for patients in the PRE-fast track group was 21.9 ± 4.14 and 22.0 ± 3.56 kg/m^2^ in the Fast-track group (*p* = 0.783) in Table 3. There was no difference between groups in the hematocrit level, the white blood cell count and the serum albumin level. More than 80% of patients had the inadequate vitamin D level in both groups.

**Table 3:** Baseline characteristics of included population

	PRE – fast track (*n* = 151)	Fast-track (n = 151)	*P* value
Age, years, mean ± SD	80.7 ± 7.51	79.7 ± 7.85	0.272
≥ 85 years, n (%)	43 (28.5)	43 (28.5)	1.000
Female, n (%)	107 (70.9)	113 (74.8)	0.438
BMI, kg/m2, mean ± SD	21.89 ± 4.14	22.01 ± 3.56	0.783
< 18.5	35 (23.2)	23 (15.2)	0.349
18.5–22.9	62 (41.1)	76 (50.3)	
23.0–24.9	20 (13.2)	21 (13.9)	
25.0–29.9	29 (19.2)	28 (18.5)	
≥ 30	5 (3.3)	3 (2.0)	
Comorbidities, n (%)			
Diabetes mellitus	57 (37.7)	52 (34.4)	0.549
Hypertension	120 (79.5)	119 (78.8)	0.887
Ischemic heart disease	25 (16.6)	21 (13.9)	0.522
Congestive heart failure	4 (2.6)	4 (2.6)	1.000
Cerebrovascular disease	34 (22.5)	38 (25.2)	0.589
COPD, Bronchiectasis or Asthma	9 (6.0)	5 (3.3)	0.274
CKD Stage 3–5	33 (21.9)	36 (23.8)	0.681
ESRD on HD/CAPD	5 (3.3)	6 (4.0)	0.759
Dementia	36 (23.8)	56 (37.1)	0.012
Charlson Comorbidity Index (CCI), n (%)			0.494
2	7 (4.6)	6 (4.0)	
3	10 (6.6)	18 (11.9)	
4	38 (25.2)	28 (18.5)	
5	26 (17.2)	34 (22.5)	
≥ 6	70 (46.4)	65 (43.1)	
Education, years, n (%)			0.479
≤ 4	95 (62.9)	89 (58.9)	
> 4	56 (37.1)	62 (41.1)	
Polypharmacy, n (%)			0.120
< 5	32 (21.2)	34 (22.5)	
5–10	64 (42.4)	76 (50.3)	
> 10	55 (36.4)	41 (27.2)	
BADL, Dependent, n (%)	21 (13.9)	22 (14.6)	0.869
IADL, Dependent, n (%)	51 (33.8)	59 (39.1)	0.339
Hematocrit, %, mean ± SD	34.71 ± 6.02	34.95 ± 5.52	0.711
WBC count, cell/mm3, mean ± SD	10,059.00 ± 3306.11	10,525.56 ± 3686.88	0.249
Serum albumin (pre-op), g/dl, mean ± SD	3.74 ± 0.54	3.76 ± 0.50	0.707
Vitamin D level, ng/mL, mean ± SD (*n* = 284)	*n* = 136 18.38 ± 9.84	*n* = 148 19.36 ± 9.42	0.393
≥ 30	16 (11.8)	18 (12.2)	0.211
20–29^a^	31 (22.8)	47 (31.8)	
< 20^b^	89 (65.4)	83 (56.1)	

*BADL* Basic activities of daily living

*IADL* Instrumental activities of daily living

^a^ vitamin D insufficiency 20–29 ng/mL

^b^ vitamin D deficiency < 20 ng/mL

Patients in both groups had similar fracture types including femoral neck fractures and intertrochanteric fractures, which were the majority types. Almost all included patients underwent surgical treatment (93.4% VS 94.0% *p* = 1.000) (Table 4). After the Fast-track program for Acute Geriatric Hip Fractures was implemented, 80.3% of patients had surgery within 72 h compared to 44.7% of those in the PRE-fast track group (*p* < 0.001). Ninety (63.4%) of the Fast-track group had surgery within 48 h compared to 39 (27.7%) of those in the PRE-fast track group (p < 0.001) (Table 4). With regard to the quality indicators for the implementation of the Fast-track program for Acute Geriatric Hip Fractures, all structure indicators were successfully arranged. For process indicators, most indicators have been achieved at a higher proportion compared to that in the PRE-fast track group as shown in Tables 4 and 5.

**Table 4:** Fracture type, time to surgery and consultation

	PRE – fast track (n = 151)	Fast-track (*n* = 151)	*P* value
Fracture Type, n (%)			0.725
Femoral neck fracture	74 (49.0)	79 (52.3)	
Intertrochanteric fracture	75 (49.7)	69 (45.7)	
Subtrochanteric fracture	2 (1.3)	3 (2.0)	
Pathologic fracture, n (%)	1 (0.7)	1 (0.7)	1.000
Surgery, n (%)	141 (93.4)	142 (94.0)	1.000
Time to surgery^a^, n (%) (*n* = 283)			< 0.001
≤ 24 h	18 (12.8)	45 (31.7)	
25–48 h	21 (14.9)	45 (31.7)	
49–72 h	24 (17.0)	24 (16.9)	
> 72 h	78 (55.3)	28 (19.7)	
Type of surgery, n (%) (n = 283)			
Arthroplasty	61 (43.3)	61 (43.0)	
Internal fixation	78 (55.3)	81 (57.0)	
Others	2 (0.7)	0 (0.0)	
Ward, n (%)			
General ward	56 (37.1)	58 (38.4)	0.812
Consult Geriatrics, n (%)	145 (96.0)	151 (100.0)	0.030
Consult within 24 h., n (%)			
Geriatrics	101 (66.9)	139 (92.1)	< 0.001
Acute pain service (APS)	6 (4.0)	47 (31.1)	< 0.001
Physical therapists (PT)	2 (1.3)	5 (3.3)	0.448

^a^Time to surgery = Admission time to operation time

**Table 5:** Rehabilitation interventions (maximum capacity) on D1 and D3 after surgery

		PRE – fast track (*n* = 141)	Fast-track (*n* = 142)	*P* value
Day 1	ROM/Ankle pumping	16 (11.3)	28 (19.7)	0.144
n (%)	Side sitting	28 (19.9)	37 (26.0)	0.446
	Standing	2 (1.4)	4 (2.8)	0.711
	Walk	12 (8.5)	18 (12.7)	0.511
Day 3	ROM/Ankle pumping	3 (2.1)	6 (4.2)	0.586
n (%)	Side sitting	16 (11.3)	22 (15.5)	0.576
	Standing	5 (3.5)	6 (4.2)	0.931
	Walk	39 (27.6)	66 (46.5)	0.005

A higher proportion of patients in the Fast-track group obtained the ambulation program on the first postoperative day. The program included the range of motion exercise (ROM) and the ankle pumping. On the 3rd post-operative day, more than 50% of patients were able to stand or walk in the Fast-track group compared to only 31% of those in the PRE-fast track group in Table 5.

The proportion of participants with delirium at any point postoperatively was similar in both groups. The percentage of delirium in the PRE-fast track group and the Fast-track group were 34.0 and 31.0% respectively (*p* = 0.583) in Table 6. Other complications were similar in both groups. Importantly, there was a higher proportion of people diagnosis of dementia in the Fast-track group that may have contributed to clinical outcomes. Therefore, a stratified analysis by dementia status was performed, which revealed a nonsignificant trend toward reduced delirium after implementing the Fast-track program among patients with dementia in Table 7.

**Table 6:** The primary outcomes

	PRE – fast track (n = 151)	Fast-track (n = 151)	*P* value
Post-operative complication, n (%)	*n* = 141	*n* = 142	
Delirium	48 (34.0)	44 (31.0)	0.583
Urinary tract infection	21 (14.9)	30 (21.1)	0.173
Pressure injury/ IAD	18 (12.8)	17 (12.0)	0.839
Pneumonia	10 (7.1)	12 (8.5)	0.670
Stroke/TIA	1 (0.7)	1 (0.7)	1.000
Myocardial infarction	1 (0.7)	2 (1.4)	1.000
Deep vein thrombosis	0 (0.0)	1 (0.7)	1.000
Pulmonary embolism	0 (0.0)	1 (0.7)	1.000
Pain score > 4	38 (27.0)	34 (23.9)	0.561
Anemia (Blood transfusion)	72 (51.1)	73 (51.4)	0.954
Wound infection	0 (0.0)	0 (0.0)	–
Delirium Type (*n* = 92), n (%)			
Hyperactive delirium	36 (75.0)	34 (77.3)	
Hypoactive delirium	5 (10.4)	0 (0.0)	
Mixed type	7 (14.6)	10 (22.7)

**Table 7:** The primary outcomes stratified by dementia

	Dementia			Non-Dementia		
	PRE - FT	FT	*P* value	PRE - FT	FT	*P* value
Post-operative complication, n (%)						
Delirium	25 (78.1)	29 (58.0)	0.061	23 (21.1)	15 (16.3)	0.387
Pneumonia	3 (9.4)	5 (10.0)	0.926	7 (6.4)	7 (7.6)	0.742
Urinary tract infection	9 (28.1)	16 (32.0)	0.710	12 (11.0)	14 (15.2)	0.376
Pressure injury/ IAD	7 (21.9)	7 (14.0)	0.355	11 (10.1)	10 (10.9)	0.857
Stroke/TIA	0 (0.0)	1 (2.0)	0.421	1 (0.9)	0 (0.0)	0.357
Myocardial infarction	1 (3.1)	1 (2.0)	0.747	0 (0.0)	1 (1.1)	0.275
Deep vein thrombosis	0 (0.0)	1 (2.0)	0.421	0 (0.0)	0 (0.0)	–
Pulmonary embolism	0 (0.0)	1 (2.0)	1.000	0 (0.0)	0 (0.0)	–
Pain score > 4	6 (18.8)	10 (20.0)	0.889	32 (29.4)	24 (26.1)	0.606
Anemia (Blood transfusion)	19 (59.4)	31 (62.0)	0.812	53 (48.6)	42 (45.7)	0.674

The secondary outcomes were summarized in Table 8. The length of stay in the Fast-track group was significantly shorter (11 (8–17) VS 13 (9–18), *p* = 0.017). However, there was no significant difference between hospital mortality and long term mortality. Most patients in both groups were discharged to home. The readmission rates in both groups were similar. The information about the patients’ function (Barthel index) at day 4, month 3 and month 12 after the operation was collected. The Barthel index of patients in both groups was subsequently improved after the patients were discharged to home, but there was no difference in the Barthel index between group in Table 8.

**Table 8:** The secondary outcomes

	PRE – Fast track (*n* = 151)	Fast-track (*n* = 151)	*P* value
Re – operation, n (%)	1 (0.7)	2 (1.4)	1.000
Length of stay, Median (25–75)	13 (9.75–18)	11 (8–17)	0.017
In-hospital mortality, n (%)	5 (3.3)	4 (2.6)	0.735
Discharge ambulation status, n (%)			0.326
Wheelchair	25 (17.1)	31 (27.1)	
Walker	112 (76.7)	112 (76.2)	
Crutch	2 (1.4)	0 (0.0)	
Unable to ambulate	7 (4.8)	4 (2.7)	
Destination of discharge, n (%)			0.223
Home	143 (97.9)	144 (98.0)	
Nursing home	1 (0.7)	3 (2.0)	
Refer	2 (1.4)	0 (0.0)	
Re – admission in 1 yr., n (%)	*n* = 146	*n* = 147	0.445
≤ 90 days	9 (6.1)	15 (10.2)	
> 90 days	18 (12.3)	20 (13.6)	
Mortality, n (%)			
3 months	8 (5.3)	9 (6.0)	0.803
6 months	12 (7.9)	16 (10.6)	0.427
1 year	14 (9.3)	20 (13.2)	0.275
Function (Barthel index), Median (25–75)			
Post op day 4 (*n* = 275)	45 (25.00–66.25)	50 (30.00–60.00)	0.617
3 months (*n* = 250)	80 (65.00–95.00)	90 (65.00–100.00)	0.100
12 months (*n* = 264)	90 (65.00–100.00)	100 (65.00–100.00)	0.066

## Discussion

This study has demonstrated the outcome of implementation of a multidisciplinary team for caring of older people with hip fractures in a large referral center university hospital in resource limited settings. The implementation was successful for accelerating the operation time and reducing length of stay. However, benefits on patient-centered outcomes were not demonstrated in this analysis contrast to several existing evidences [13, 34]. This finding might stem from several factors.

Outcomes including in-hospital complications after implementation of the Fast-track program for Acute Geriatric Hip Fractures were analyzed. There was no statistical difference in the incidence of delirium between before and after implementation of the program. Published studies reported reductions in post-operative medical complications, delirium and in-hospital mortality mainly occurring in settings with applied routine geriatric consultation [10, 12, 13, 17, 19, 21, 35, 36]. In Siriraj hospital, geriatric consultation was a common practice (96%) before the Fast-track program for Acute Geriatric Hip Fractures was implemented. This may have contributed to the inability to detect important differences following implementation of the program. Moreover, the overall incidence of post-operative delirium in the study was approximately 32%, lower than the 45% rate reported by a previous study in a similar Thai population [37] and lower than the rates reported by studies in other countries [10, 38–41]. This might indicate that the geriatric consultation in the PRE-fast track era was a standard level of care for a decrease in delirium. The study has affirmed the geriatric consultation also playing an important role in delirium reduction in resource-limited countries.

The prevalence of dementia in this study was significantly higher in the Fast-track group (37.1% VS 23.8%, *p* < 0.012) and higher than that reported by earlier studies [12, 35, 37, 40]. Dementia is an established risk factor for post-operative complications and outcomes including delirium, rehabilitation achievement and functional outcomes after discharged [40, 42], so a post-hoc analysis according to dementia status was performed. The analysis revealed a non-significant trend of delirium reduction for patients in both groups, with dementia and without dementia, after the program was implemented. The incidence of pressure injury was also lower in the people living with dementia in the Fast-track group. This makes clinical sense because delirium and pressure injury may be a result of inadequate pain control and prolonged immobilization, commonly experienced in people living with dementia. The program with multidisciplinary team was designed to prevent these undesirable symptoms.

The incidences of pneumonia, urinary tract infection, and pressure injury were higher than those in the previous studies [13, 17, 43] and were not reduced after the Fast-track program for Acute Geriatric Hip Fractures had been implemented. Early ambulation is considered one of the most valuable postoperative strategies to reduce postoperative pneumonia and pressure injury [26, 44]. Early ambulation remained suboptimal, although was more common in the Fast-track group, but the statistically significant reductions in post-surgical complications were not observed. Barriers could possibly stem from the attitudes of involved healthcare staff, availability of therapists, or the patients’ condition. A more comprehensive plan of barrier reduction from all involved parties with more practical strategies to apply the ambulation program might improve engagement and outcomes.

Moreover, during the first few months of the Fast-track program for Acute Geriatric Hip Fractures, the APS team did not fully operate and some patients might not obtain the optimal pain control. Complications such as delirium and inadequate rehabilitation were also more common before the APS was fully implemented.

Hip fractures in older people affect short term mobility and long term functional outcomes [45–47]. The functional outcomes were measured by using the Barthel index. Although, there was no significant difference in the Barthel index for all comparisons between groups but there was a trend toward better function in the Fast-track group. Moreover, most patients achieved improved functional outcomes over time and the majority reached full mobility at 1 year, which has been better results than other study [48]. Discharge destination might contribute to functional status of the patients. In other studies, most of the patients were discharged to institutional care units while most of the patients in this study were discharged back to home because of cultural norms for care of older people in Thailand. After discharged to home, the patients were looked after by dedicated family, which could contribute to better functional outcomes.

Debate on time to surgery for hip fracture surgery remains, not for the benefit of expedited surgery but rather the optimal time of how fast the time should be [23, 49, 50]. Most studies appear to indicate the ‘optimal time’ at 48 h following the cut off points utilized in meta-analyses [50]. Some important difficult-to-measure confounding factors might be the patients’ risk for experiencing medical complications and other negative clinical consequences and the accompanied care provided in different settings. Patients included in the study, conducted in a large referral center, have more co-morbidities with higher Charlson comorbidity index compared to previous studies [17, 51]. However, in this study, there was a similar rate of medical complications for the whole cohort compared to other studies in other settings. This might be one reason why we could not demonstrate the difference in primary outcome as we have reached the ceiling for reducing some complications. Accordingly, there were rooms for improving the program shown in the results. The rate of involvement of main specialties in the program remain low in some context and might contribute to the results observed. In the present cohort, the decisions to undergo surgery were based on agreement of all involved specialists for each patient. The delayed time to surgery might be to optimize medical conditions for the patients. To achieve the ultimate goal for the Fast-track program for Acute Geriatric Hip Fractures, we should focus on the orchestrating implementation of all involved disciplines, as well.

### Strengths and limitations

Our study has several strengths. Data for this study was collected from several sources, including a prospective registry (FLS) allowing us to monitor several long-term outcomes such as functional status and quality of life. Data regarding related medical complications and possible interventions were thoroughly explored to identify the gap of practice. The selection of consecutive cases would reduce the selection bias for this study. Limitations of the study are mainly stemmed from its retrospective design. The retrieval of information from medical records might underestimate some interventions and complications. Delirium ascertainment is one important limitation as it is usually under-recognized and there was no routine delirium screening in the hospital. Nevertheless, several measurements were taken to ensure that most cases of delirium could be identified. Several interventions such as physiotherapy program could be underestimated. Moreover, this is a single center study in a university hospital, the generalizability of the results might be an issue.

## Conclusion

We demonstrated the feasibility of the implementation of the comprehensive multidisciplinary care team in Siriraj hospital, a large referral center and a university hospital with complex administrative structures in resource-limited setting. The Fast-track program for Acute Geriatric Hip Fractures reduced the length of hospital stay and time to surgery. Moreover, there was no increase in medical complications. However, several interventions in the program were not implemented as planned. A better embarking rate of all interventions might improve the mortality rate in this group of population.

## Data Availability

The datasets used and/or analysed the current study are available from the corresponding author on reasonable request.
